# Ultrasound-guided erector spinae plane block versus rhomboid intercostal sub-serratus plane block for postoperative analgesia in open radical nephrectomy: a randomized clinical study

**DOI:** 10.1186/s12871-025-03377-4

**Published:** 2025-10-22

**Authors:** Doaa Abd Eltwab, Sayed M. Abed, Ahmad Saad, Maha A. Abdel Aliem, Khaled A. Elsamahy, Fatma H. Elshamy, Ahmed F. Gad, Walaa Y. Elsabeeny

**Affiliations:** Department of Anesthesia, Surgical Critical care and Pain management, National Cancer Institute, Cairo University, Kasr Al Eini Street, Fom El Khalig, Cairo, 11796 Egypt

**Keywords:** Nephrectomy, Regional anesthesia, Analgesia, Morphine

## Abstract

**Background:**

Open radical nephrectomy often results in significant acute postoperative pain. Regional anesthesia offers an alternative analgesic approach in these situations. This study aims to assess and compare the effectiveness of ultrasound-guided rhomboid intercostal sub-serratus (RISS) block with Erector Spinae Plane Block (ESPB) in patients undergoing open radical nephrectomy.

**Methods:**

This randomized clinical trial included 42 patients scheduled for open radical nephrectomy (RN). Patients were randomly assigned to one of two groups: the ESPB Group (*n* = 21), which received an ESPB with 30 ml of bupivacaine 0.25%, or the RISS Group (*n* = 21), which received a RISS block with 30 ml bupivacaine 0.25%. Total morphine consumption was set as the primary outcome while pain scores, perioperative hemodynamics and time to postoperative analgesia were considered as secondary outcomes.

**Results:**

Total morphine consumption within the first 24 postoperative hours was significantly lower for the ESPB group (16.4 ± 2.5 mg) compared to the RISS group (18.2 ± 1.8 mg), *p* = 0.011. VAS pain scores at rest were significantly lower in the ESPB group at 12 and 18 h (*p* = 0.002, *p* = 0.018) respectively. VAS scores with movement were significantly lower for the ESPB group at 8 h,12 h, and 18 h (*p* = 0.011, *p* = 0.001, and *p* = 0.018 respectively). The first time to receive postoperative analgesia was significantly longer in the ESPB group (7.3 ± 2.1 h) than in the RISS group (6.0 ± 2.1 h), *p* = 0.048. Both groups were comparable in the number of PCA boluses, the number of patients requiring intraoperative fentanyl increments, or recovery time.

**Conclusion:**

Ultrasound-guided ESPB provides slightly superior postoperative analgesia compared with RISS block in patients undergoing open radical nephrectomy via subcostal anterior incision for renal malignancies.

**Trial registration:**

The trial was registered at Clinical Trials.gov. https://clinicaltrials.gov/study/NCT05822011, trial ID (NCT05822011, 14 March 2023).

**Supplementary Information:**

The online version contains supplementary material available at 10.1186/s12871-025-03377-4.

## Introduction

According to the GLOBOCAN (Global Cancer Incidence, Mortality and Prevalence in Oncology) around 400,000 new cases of renal cell carcinoma (RCC) were diagnosed in 2020 [1]. A range of therapeutic modalities is available for the management of RCC, contingent upon the disease stage at diagnosis. Radical nephrectomy is the primary therapeutic approach for RCC. The advancement of this therapy has significantly reduced RCC-related mortality in recent decades [2].

Open radical nephrectomy is linked to considerable acute pain and necessitates opioid analgesia if localized analgesia is either bypassed or proves ineffective [3]. Thoracic epidural analgesia is commonly employed to manage this pain. However, neuraxial methods are associated with many drawbacks, such as hypotension due to bilateral sympathectomy, in addition to certain contraindications [4]. Regional anesthesia is a tempting alternative in these cases. It significantly contributes to acute pain management and the reduction of systemic opioid consumption and its related adverse effects [5].

The Erector Spinae Plane Block (ESPB) is a straightforward regional practice that utilizes readily identifiable sonographic features [6]. Some studies have examined the application of ESPB in nephrectomy [7–10]. It can offer superior analgesia for an extended duration, resulting in reduced pain scores and diminished intraoperative and postoperative opioid usage compared to patient-controlled analgesia [11].

Another locoregional analgesic technique that works on analgesia for the sub-serratus and intercostal areas is the rhomboid intercostal sub-serratus (RISS) block. This innovative approach improves the dissemination of local anesthetics (LA), resulting in superior pain management across a wider region than traditional blocks [12]. It is generally indicated for patients undergoing abdominal surgeries, including laparoscopic procedures, open cholecystectomy, and hernia repairs, where effective postoperative acute pain control is crucial [12, 13].

This study aimed to compare the efficacy of ultrasound-guided RISS block and ESPB for acute pain management in patients undergoing open radical nephrectomy for the treatment of renal cell carcinoma.

## Methods

Following the institutional review board of National Cancer Institute and Department Of Anesthesia, Surgical Critical Care and Pain Management ethical committee approval, this randomized trial was carried out between April 2023 and May 2025 at National Cancer Institute, Cairo University, Egypt. Written informed consent was obtained from each participant. Data confidentiality was strictly maintained. The study adhered to the CONSORT reporting guidelines and was conducted in compliance with national institutional ethical standards and the ethical principles of the Declaration of Helsinki. The study was registered in clinical trial. gov https://clinicaltrials.gov/study/NCT05822011, trial ID (NCT05822011, 14 March 2023). Forty-two patients planned for open radical nephrectomy operation with subcostal incision were enrolled into the study. The inclusion criteria were age 18–65 and physical status ASA II or III. The exclusion criteria included patient refusal, compromised cardiac or respiratory conditions, kidney or liver function impairment, a history of chronic pain medication use, and a body mass index (BMI) exceeding 35 kg/m². Additionally, patients with septicemia, local infections, impaired platelet count or function, and abnormal coagulation profiles were also excluded.

Patients were enrolled into two equal groups using computer-generated random sequence. The generated numbers were then concealed in closed envelopes. The first group received ESPB (*n* = 21) while the second group received RISS block (*n* = 21), both blocks were done under ultrasound guidance. The study was designed as a double-blinded study where the outcome investigator and patients were blinded to the block applied. Outcome assessment was conducted by both the intraoperative anesthetist and the anesthesia resident assigned to postoperative follow-up. To preserve patient blinding, participants were notified that a local anesthetic would be administered before needle insertion, but no specifics were provided regarding the number or site of injections.

### Study protocol

Eligible patients underwent history taking, physical examination, laboratory investigations, and radiological imaging in the preoperative anesthesia assessment clinic. Additionally, the use of VAS was explained to the patients, and they were trained on how to report their pain score, where 0 was used when there was no pain and 100 for severe pain.

In the holding area, patients’ vital signs were monitored using standard ASA monitors (ECG, NIBP, peripheral arterial oxygen saturation). Then an IV cannula (18G) was inserted with the commencement of IV Ringer’s acetate (10–15 ml/kg/hr). All patients received IV midazolam (0.02 mg/kg) as a premedication. Then, the regional block was performed according to group assignment.

### Technique of erector spinae plane block

**Fig. 1:**
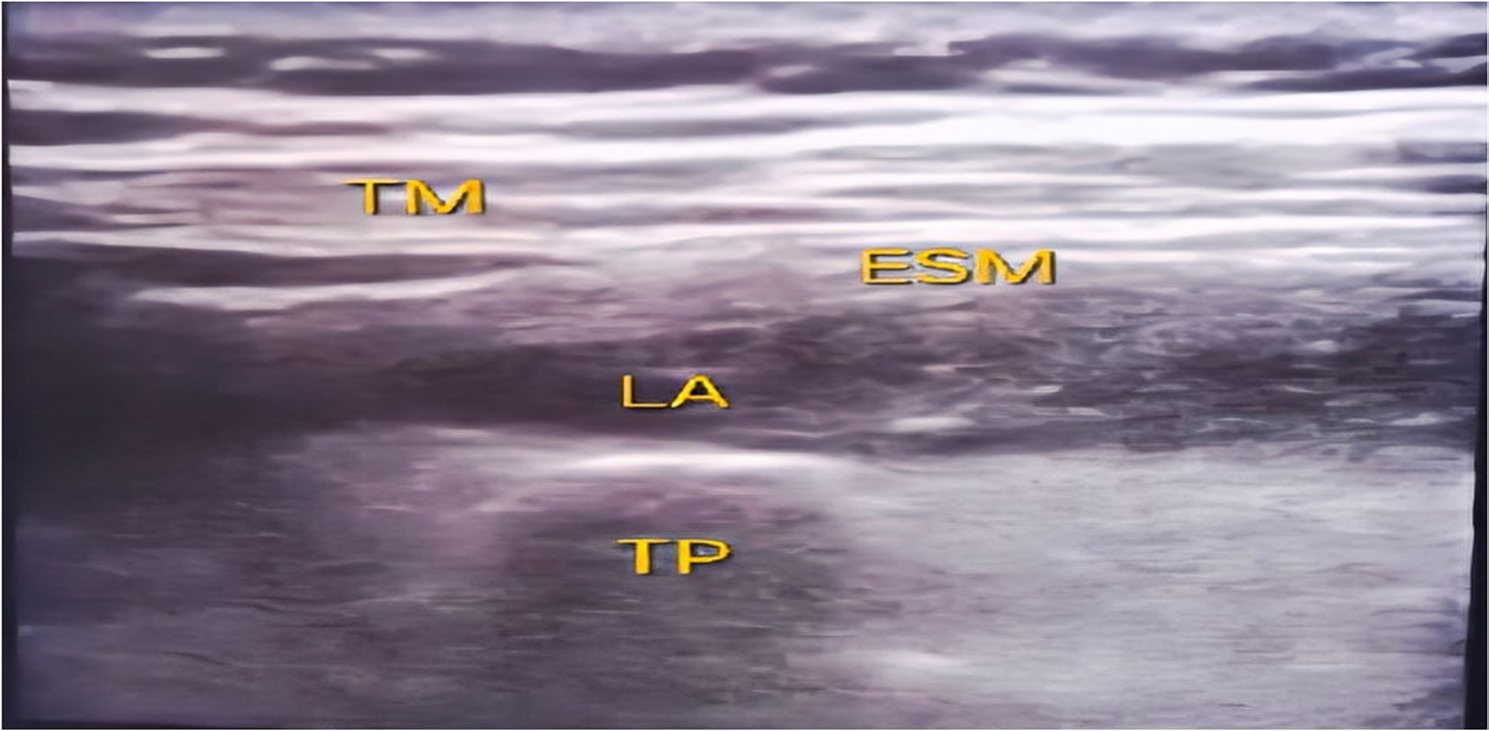
ESPB; TM: trapezius muscle, ESM: erector spinae muscle, LA: local anesthetic, TP: transverse process

In the sitting position, after draping under aseptic precautions, the block was done using (Fujifilm Sonosite M-Turbo Ultrasound system), first an ultrasound scan was done in parasagittal orientation to identify the transverse process of the 8th thoracic vertebra. Once located, and after identification of the erector spinae and trapezius muscles, local anesthesia was infiltrated at the entry site then an 18-gauge epidural needle was advanced in a cranio-caudal direction using an in-plane technique to reach the transverse process. After needle-transverse process contact 2 ml of normal saline was injected “under real-time visualization” of saline spread to confirm correct position. Then, 30 ml of bupivacaine 0.25% was injected after negative aspiration of blood [14], (Fig. 1).

### Technique of rhomboid intercostal sub-serratus plane block

**Fig. 2:**
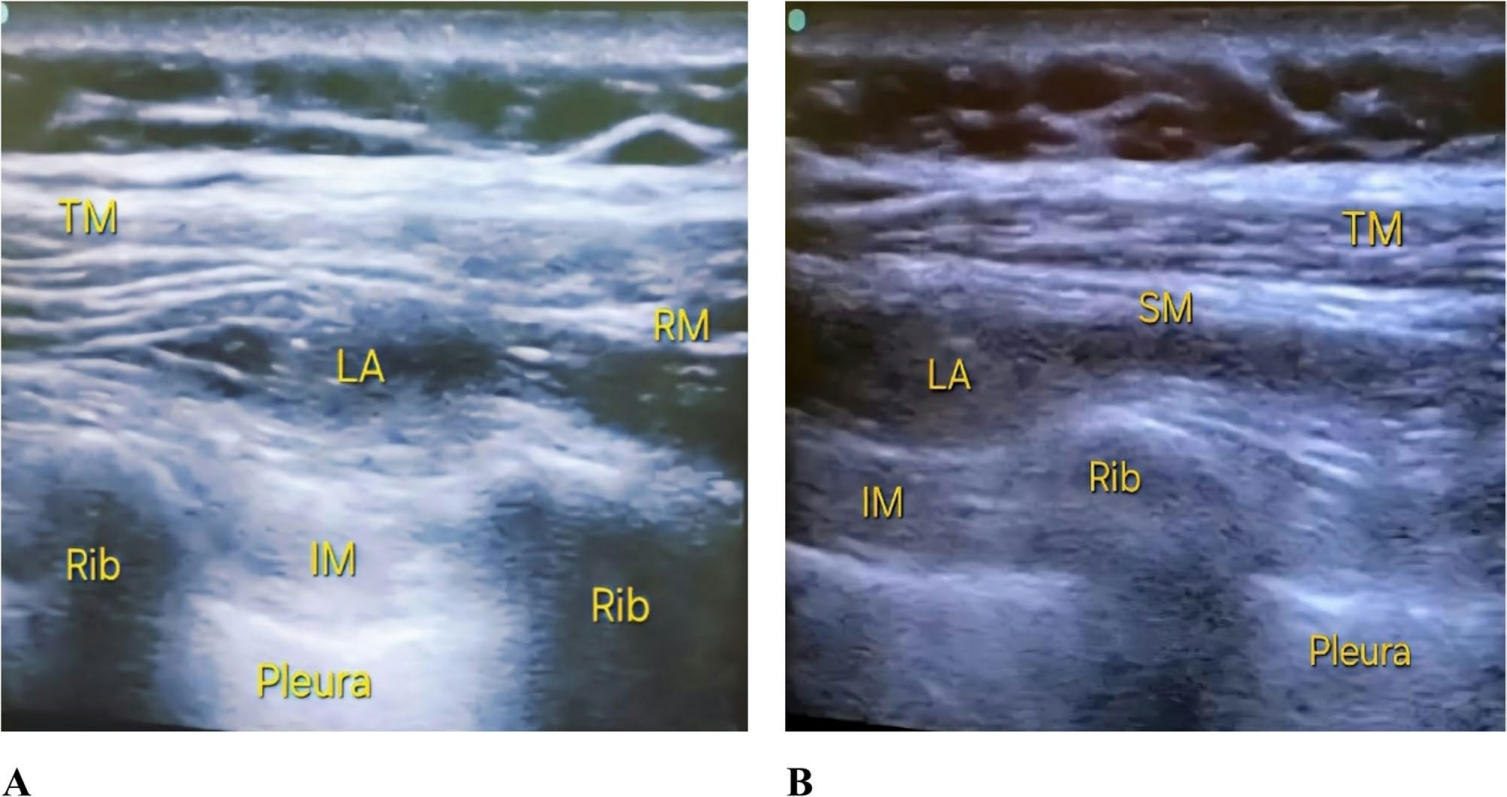
(**A**) Rhomboid intercostal block; TM: trapezius muscle, RM: rhomboid muscle, LA: local anesthetics, IM: intercostal muscles (**B**) Sub serratus block; TM: trapezius muscle, SM: serratus muscle, LA: local anesthetic, IM: Intercostal muscles

In the sitting position, after draping under aseptic precautions, the block was done using (Fujifilm Sonosite M-Turbo Ultrasound system). First, an ultrasound scan was done in a sagittal orientation to identify the 5th thoracic spinous process as a landmark. A high-frequency (6–12 MHz) linear ultrasound probe was positioned adjacent to the medial border of the scapula. The probe was then rotated slightly, placing it 1–2 cm medial to the scapular border, allowing for a clear visualization of the anatomical layers. The plane between the rhomboid major muscle and intercostal muscles was then located. The skin overlying the site of needle insertion was anesthetized using 2 ml of 1% lidocaine. An 18-gauge epidural needle was then advanced in an in-plane technique, from a superomedial to an inferolateral direction. At the T5 level, 15 ml of bupivacaine 0.25% was carefully injected. Subsequently, the probe was advanced in a caudal and lateral direction to visualize the tissue plane between the serratus anterior and intercostal muscles at the level of T7-T8 ribs. At this point, another 15 ml of bupivacaine 0.25% was injected to complete the block [12, 15], (Fig. 2A, B).

At the end of the block a lung ultrasound scan was performed to exclude pneumothorax [16]. Twenty minutes after the block, the degree of sensory block was assessed by the pinprick test along the anterior axillary line from T5 to T10 dermatomes. When the pinprick was recognized as a touch by a blunt object or not recognized at all, this was considered a successful block; otherwise, the patient was excluded from the study.

### Anesthetic management

At the operating theatre, all patients were kept under monitoring using standard ASA monitors. Anesthesia was induced using fentanyl 2 µg/kg and propofol 1–2 mg/kg and 0.6 mg/kg IV of rocuronium. General anesthesia was maintained with sevoflurane inhalation, titrated to a MAC level between 1.0 and 1.3 and supplemental doses of rocuronium. Ventilation rate and tidal volume were adjusted to maintain an end-tidal CO₂ level between 30 and 35 mmHg. All the patients received intravenous infusion of paracetamol 1 gm and ketorolac 30 mg as a part of multimodal analgesia. In case of elevated mean arterial pressure (MAP) or heart rate (HR) above 20% of baseline levels and after the exclusion of lack of anesthesia a rescue analgesic dose of fentanyl 1 µg/kg was given, then total amount of intraoperative fentanyl was recorded. Perioperative hemodynamics (MAP and HR) were continuously monitored and recorded. At the end of surgery, neostigmine (0.05 mg/kg) and atropine (0.02 mg/kg) were given to reverse any residual muscle relaxant effect followed by extubation. Recovery time (from discontinuing sevoflurane to reaching an Aldrete score ≥ 9) was recorded.

After patients were transferred to the post-anesthesia care unit (PACU), MAP, HR and VAS scores were recorded. Postoperatively, in PACU and for the first 24 h MAP, HR and VAS scores were recorded at 4, 8, 12, 18 and 24 h, intravenous morphine (3 mg) was administered upon patient-reported pain with a VAS score ≥ 40. This was immediately followed by the initiation of patient-controlled analgesia (PCA), consisting of 20 mg morphine and 1 mg granisetron diluted in 100 ml saline, delivered at a basal rate of 4 ml/hour with a 1 ml bolus and a 15-minute lockout interval. This regimen was designed to maintain VAS scores ≤ 40 and ensure timely relief. Additionally, paracetamol 1gm every 8 h were infused as a part of multimodal analgesia. The total amount of morphine consumed in 24 h was recorded. Patient satisfaction was evaluated on a 1–5 Likert scale, where ratings of 1 represent dissatisfaction, while ratings of 5 denote extreme satisfaction.

Opioid side effects such as sedation,nausea, vomiting, and respiratory depression (respiratory rate <10 per minute) were recorded.For patients who experienced multiple episodes of vomiting, IV ondansetron was administeredat a dose of 0.1 mg/kg.

The primary outcome measure was the total amount of morphine consumed in the first 24 h postoperatively. Secondary outcomes were changes in hemodynamics, the need for intraoperative fentanyl increments, time of first request of analgesia, VAS scores, recovery time, patient satisfaction, block-related complications (e.g., local anesthetic toxicity, hematoma formation, and pneumothorax), and nausea and vomiting.

### Sample size estimation

Because no similar studies had been done, a preliminary study was conducted with ten individuals in each group to determine the effect size. The effect size was estimated by calculating morphine consumption during the first 24 postoperative hours. The mean consumption was 17.4 ± 2.65 mg and 15.2 ± 2.25 mg in the RISS and ESPB groups, respectively.

Based on these values, the study would require a sample size of 21 for each group (i.e. a total sample size of 42, assuming equal group sizes), to achieve a power of 80% and a level of significance of 5% (two sided), for detecting a true difference in means between the RISS and the ESPB group of 2.2 mg. Although the calculated sample size was 21 participants per group, it was increased by 15% (to 24 per group) to preserve statistical power in the event of participant attrition.

### Statistical analysis

SPSS software (IBM, Armonk, New York, United States) was used for analysis and modeling of statistical data. Data was summarized and expressed as means, standard deviations or medians and ranges for quantitative data, while numbers and percentages were used to express categorical data. Comparison between the groups was done using an independent sample t-test normally distributed data and a Mann-Whitney test for non-normally distributed data. Chi-square or Fisher’s exact test were used as appropriate for comparing categorical data. All statistical tests were two-sided. A p-value 

## Results

**Fig. 3:**
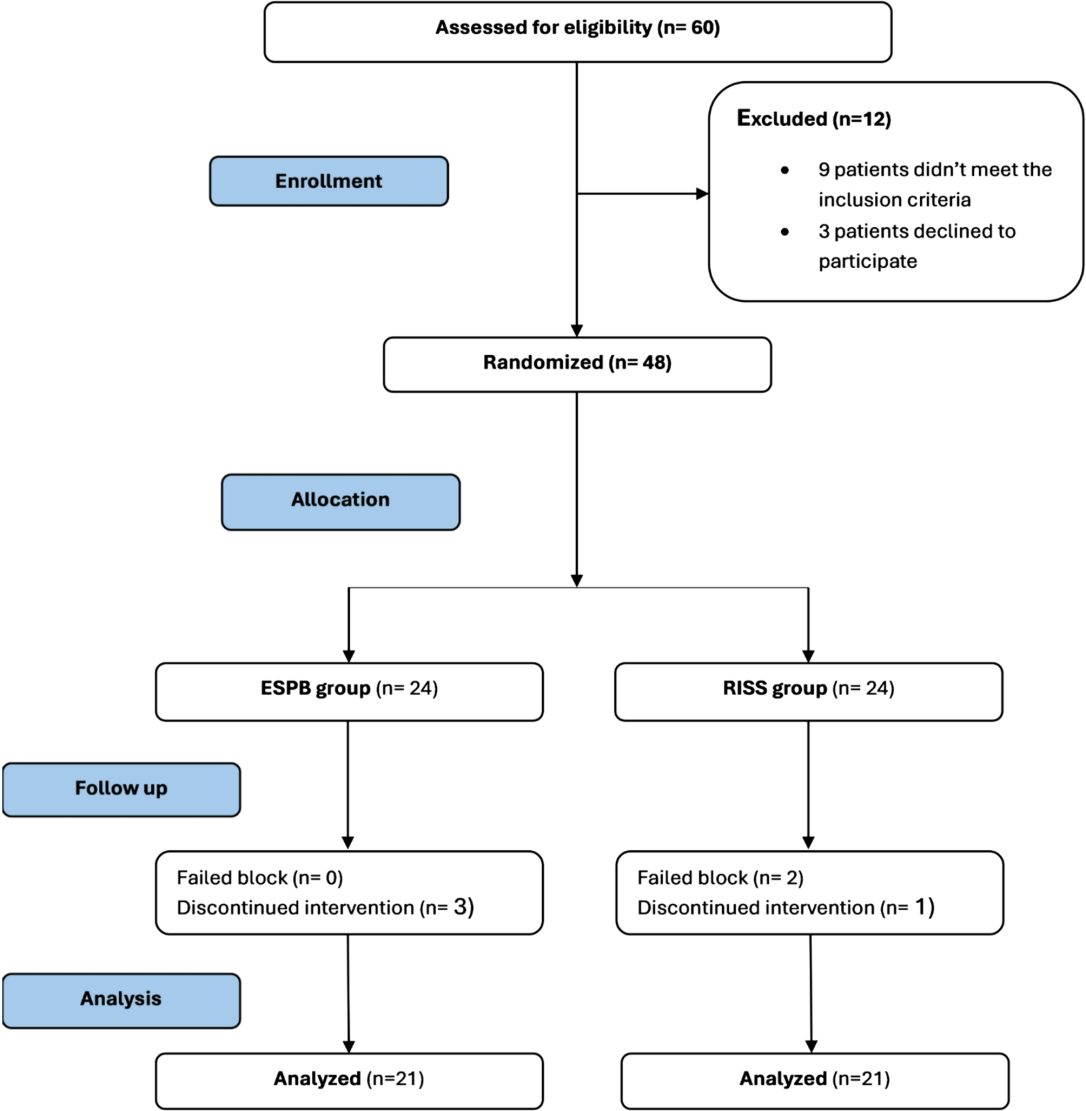
Patient flow chart

A total of 60 patients were screened for eligibility. Twelve patients were excluded, nine did not meet inclusion criteria, and three declined participation. Forty-eight patients were subsequently randomized. During follow-up, six were excluded: two due to failed blocks and four due to discontinued interventions. The remaining 42 patients were included in the final statistical analysis (Fig. 3).

Table 1 shows comparable baseline characteristics in the two groups, including age (*p* = 0.966), BMI (*p* = 0.122) and ASA class (*p* = 0.232). However, the time required to perform the regional block was significantly shorter in the ESPB group (8.0 ± 1.3 min) compared to the RISS group (15.0 ± 3.3 min; *p* < 0.001).

**Table 1:** Baseline characteristics of the two studied groups

		ESPB Group (*n* = 21)	RISS Group (*n* = 21)	*p*-value
Age (years)		51.7 ± 9.9	51.6 ± 11.5	0.966*
Weight (kg)		82.2 ± 9.7	78.6 ± 10.7	0.252*
Height (cm)		1.66 ± 0.07	1.67 ± 0.05	0.429*
Body mass index (kg/m^2^)		30.12 ± 4.02	28.2 ± 3.86	0.122*
Side	Rt	10 (47.6%)	9 (42.9%)	0.757^#^
	Lt	11 (52.4%)	12 (57.1%)	
ASA Class	II	18 (85.7%)	21 (100%)	0.232^#^
	III	3 (14.3%)	0 (0%)	
Block Duration (min)		8.0 ± 1.3	15.0 ± 3.3	<0.001*

Data are presented as mean ± SD or number (%)

*ASA* American Society of Anesthesiologists.

* t-test for independent samples

# Chi-square test

Analgesic outcomes and recovery parameters are summarized in Table 2. The ESPB group exhibited significantly lower total morphine consumption within the first 24 postoperative hours (16.4 ± 2.5 mg) compared to the RISS group (18.2 ± 1.8 mg), *p* = 0.011. Additionally, the time to first morphine rescue request was significantly prolonged in the ESPB group (7.3 ± 2.1 h) versus the RISS group (6.0 ± 2.1 h; *p* = 0.048). No significant differences were observed in the number of patients requiring intraoperative fentanyl (*p* = 0.697), the number of PCA boluses (*p* = 0.663). Recovery time was comparable between the two groups. The mean recovery time was 11.8 ± 1.3 min in the ESPB group and 12.2 ± 1.1 min in the RISS group, with no statistically significant difference observed (*p* = 0.214).

**Table 2:** Analgesic profile and recovery time of the two studied groups

	ESPB Group (*n* = 21)	RISS Group (*n* = 21)	*p*-value
The number of patients required rescue fentanyl	3 (14.3%)	5 (23.8%)	0.697^#^
The time to first request of rescue morphine analgesia (hrs.)	7.3 ± 2.1	6.0 ± 2.1	0.048*
Median time to request rescue morphine (hrs.)	8.0 (7.4–8.6)	6.0 (5.2–6.8)	0.039^$^
Postoperative morphine (mg)	16.4 ± 2.5	18.2 ± 1.8	0.011*
Number of PCA boluses	2 (1–4)	2 (1–5)	0.633^
Recovery time (min.)	11.8 ± 1.3	12.2 ± 1.1	0.214*

Data are presented as number (%), mean ± SD, or median (range)

*PCA* Patient-controlled analgesia

^#^ Chi-square test

* t-test for independent samples

^$^ Kaplan-Meier analysis

^ Mann-Whitney test

**Fig. 4:**
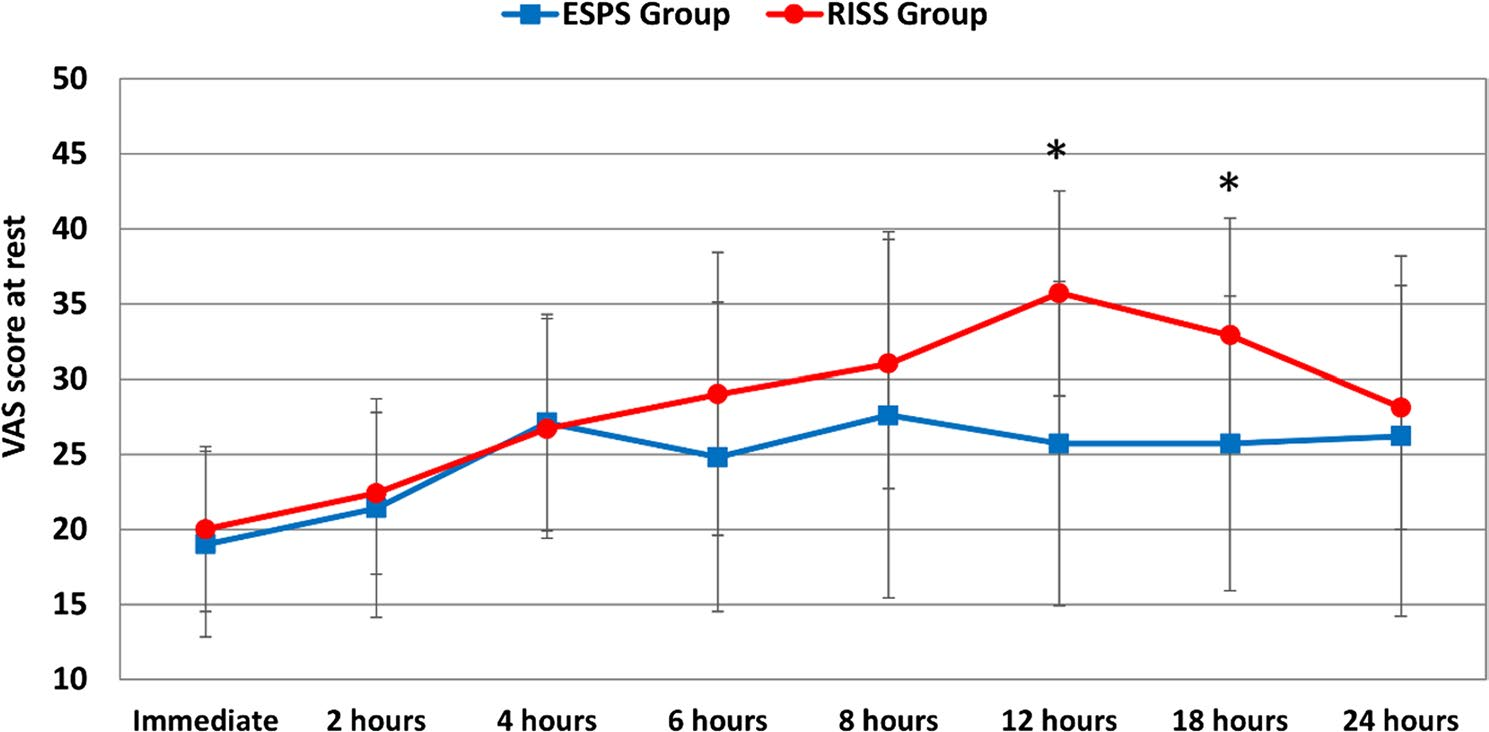
Postoperative visual analog scores at rest in the two studied groups, *: intergroup significant difference

**Fig. 5:**
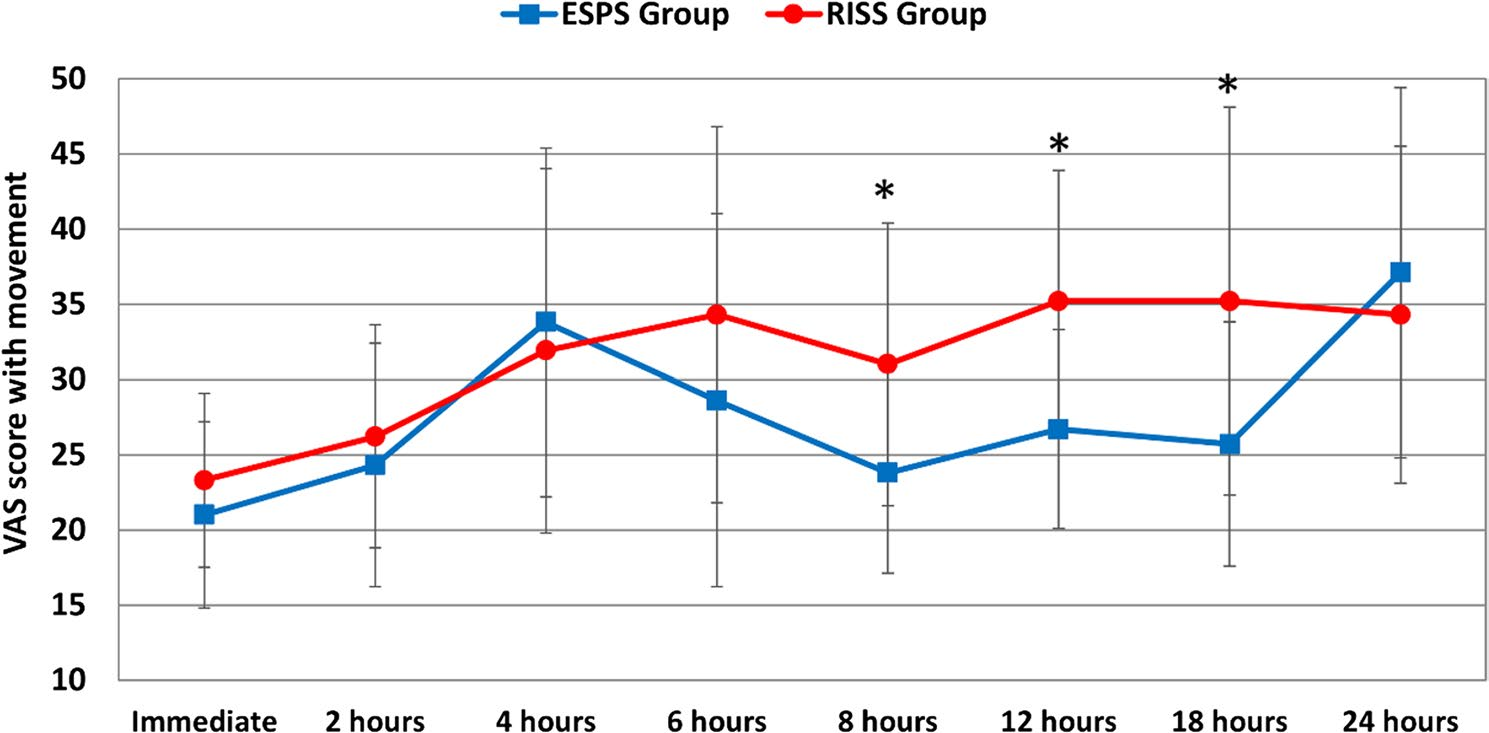
Postoperative visual analog scores with movements in the two studied groups, *: intergroup significant difference

Postoperative pain scores assessed by the VAS are detailed in Figs. 4 and 5. At rest, the ESPB group demonstrated significantly lower VAS scores at 12 and 18 h (25.7 ± 10.8 and 25.7 ± 9.8) compared to the RISS group (35.7 ± 6.8 and 32.9 ± 7.8), *p* = 0.002 and 0.018 respectively. During movement, VAS scores were significantly reduced in the ESPB group at 8, 12 and 18 h (23.8 ± 6.7, 26.7 ± 6.6 and 25.7 ± 8.1), compared to RISS group (31.0 ± 9.4, 35.2 ± 8.7 and 35.2 ± 12.9), (*p* = 0.011, 0.001 and 0.018), respectively. No statistically significant differences were detected on other points.

**Fig. 6:**
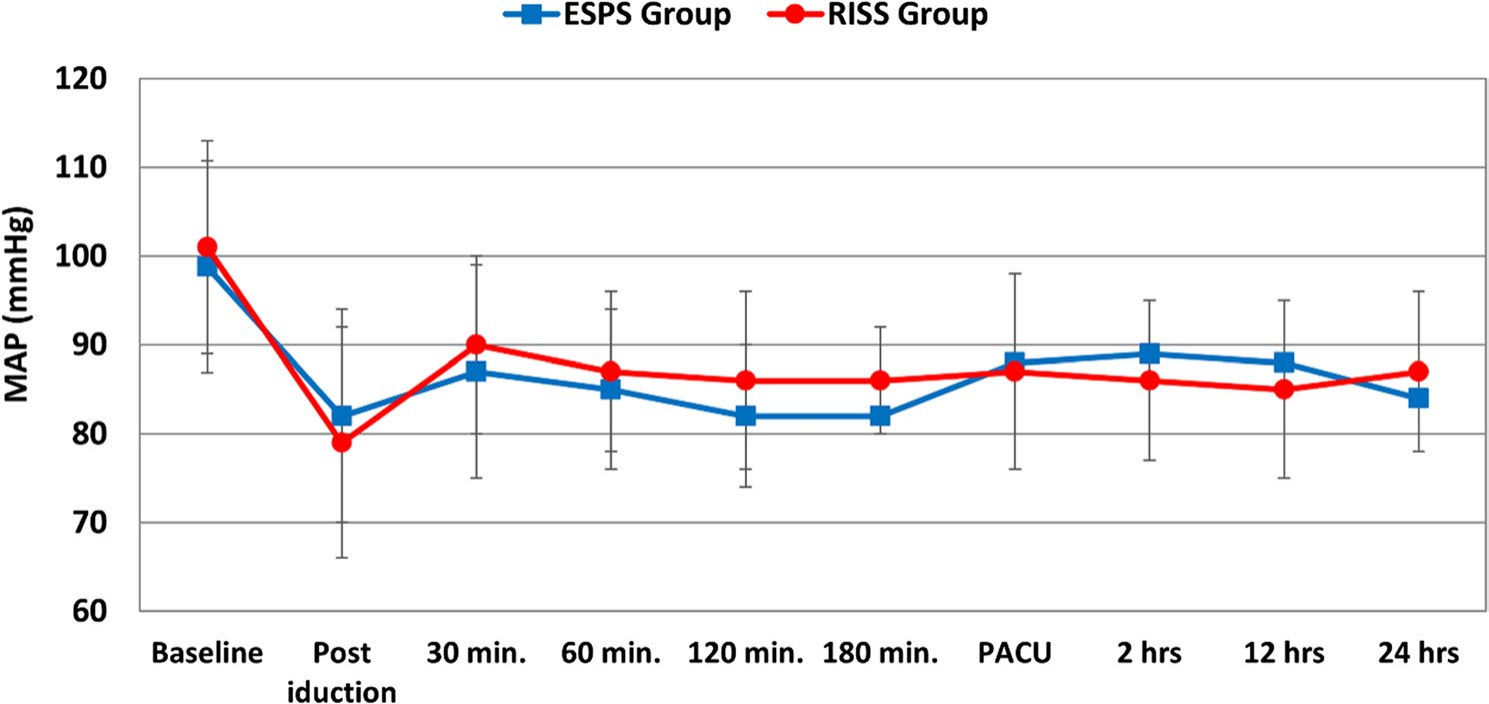
Perioperative changes of mean arterial pressure in the two studied groups

**Fig. 7:**
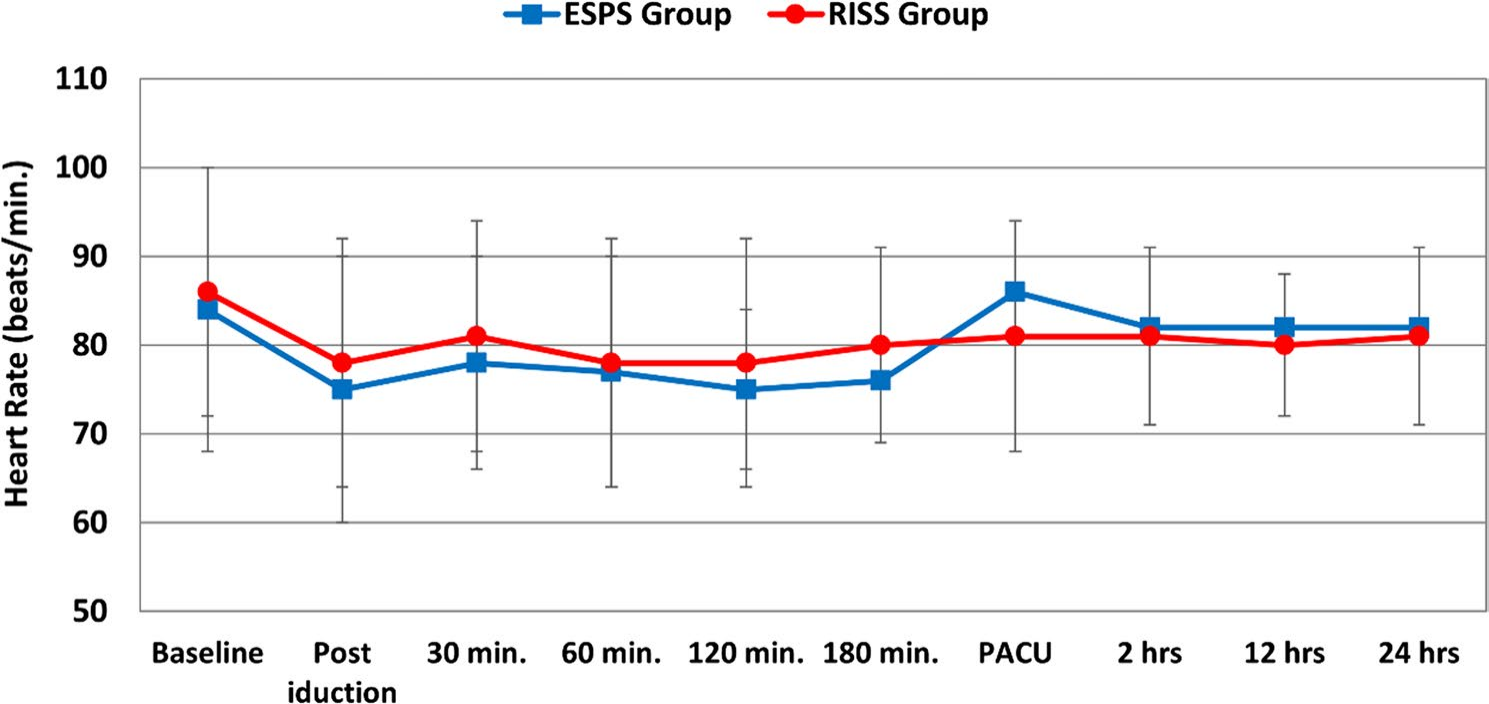
Perioperative changes of heart rate in the two studied groups

Hemodynamic variables including mean arterial pressure (MAP) and heart rate (HR) remained stable and comparable between the two groups throughout the intraoperative and postoperative periods, with no significant intergroup differences at any recorded time point (Figs. 6 and 7).

There was no significant difference between the two groups in satisfaction score (*p* = 0.098). The ESPB patients reported a median satisfaction score of 4 [3–5] compared to 4 [2–5] in the RISS patients.

Regarding side effects, the incidence of postoperative nausea and vomiting (PONV) was low and did not differ significantly between the groups. One patient in the RISS group experienced mild nausea that was resolved spontaneously, and no vomiting was reported in either group. Importantly, no serious complications such as pneumothorax, local anesthetic systemic toxicity, or hematoma were observed in any patient throughout the study.

## Discussion

This study demonstrated that ESPB resulted in lower postoperative morphine consumption within the first 24 h and a longer duration of analgesia compared to RISS following radical nephrectomy for RCC. ESPB was associated with significantly reduced pain scores at certain postoperative time points. Both techniques targeted the T5-T10 dermatomes in the two groups. Overall, both techniques maintained hemodynamic stability throughout the perioperative period.

Surgical approaches for partial or radical nephrectomy include open, laparoscopic, and robotic-assisted laparoscopic techniques [17]. Open kidney surgery is typically performed under general anesthesia, as it offers greater comfort for patients in terms of procedure duration, blood loss, and positioning on the surgical Table [18]. At our institution, open radical nephrectomy is the primary technique used for patients with RCC via a transabdominal or anterior subcostal incision.

Following open radical nephrectomy, effective analgesia is essential for early mobilization, efficient coughing, and minimizing postoperative respiratory problems. Thoracic epidural analgesia has traditionally been considered the gold standard for pain management in these cases. However, there has been a notable shift away from this neuraxial technique due to challenges with ambulation, hypotension, and excessive intravenous fluid administration [19]. As an alternative, the use of regional block techniques is encouraged for pain control following nephrectomy, which delivers focused analgesia while reducing systemic opioid consumption and its related adverse effects, including respiratory depression, nausea, and constipation [5]. The dermatomes requiring coverage by the regional block depend on the incision applied. In the transabdominal approach, the T6-T10 dermatomes should be covered [3]. Therefore, we have chosen two blocks that can cover this area in the current study.

ESPB involves dissemination of local anesthetics in the fascial plane between the vertebral transverse process and the erector spinae muscles complex. As a result, the craniocaudal fascial plane is infused with the LA [20]. Additionally, the local anesthetic can extend anteriorly into the epidural and paravertebral spaces and laterally into the intercostal regions across multiple levels. ESPB can block both visceral and somatic sensory inputs [21, 22]. It achieves selective blockade primarily through unmyelinated C fibers, rather than the larger A-δ and A-γ fibers [21]. We believe that these suggested mechanisms can explain the analgesic efficacy of ESPB in patients of the current study. The block at the level of T8 achieved a dermatomal coverage from T5 to T10 (confirmed by pinprick test), which is satisfactory for the subcostal approach used in the current series.

The utilization of ESPB for analgesia in abdominal surgery is relatively limited, with only few published studies addressing its application. Limited research has investigated the use of ESPB in nephrectomy procedures [7–10]. An initial article described lumbar ESBP for postoperative analgesia in two pediatric patients following open radical nephrectomy under general anesthesia for Wilms’ tumor. The two patients did not require rescue analgesics during the postoperative 48 h [8]. Kim et al. reported a case of intermittent ESPB as part of multimodal analgesia after open radical nephrectomy for RCC under general anesthesia via a flank incision. ESPB was performed after surgery at the T7 level. It provided sensory loss in the T2–T10. Pain scores were effectively reduced without any additional analgesics in the postoperative period [10].

One study reported comparable analgesic efficacy of ESPB versus anterior Quadratus Lumborum block (QLB) following open radical nephrectomy. The two techniques were more effective compared to a control group in terms of reduced pain scores and morphine consumption, and longer analgesic time. Both techniques had comparable analgesia. The coverage of ESPB extended from T6 to T12 [7]. Another study found ESPB as a promising, direct, and secure method for postoperative analgesia following open radical nephrectomy in renal malignancy procedures. ESPB offered superior analgesia for an extended duration, resulting in reduced pain scores and diminished intraoperative and postoperative opioid usage compared to PCA [11]. The findings of the current study support the analgesic efficacy of ESPB in patients undergoing open radical nephrectomy.

The comparator technique in the current study was the RISS block, which is notable for its dual-targeting technique, impacting both the intercostal and sub-serratus regions. This innovative approach improves the dissemination of local anesthetics, resulting in superior pain management across a wider region than traditional blocks [12]. RISS block involves blocking the lateral cutaneous branches of the intercostal nerves from T3 to T9 through the administration of LA in two fascial planes between the rhomboid and intercostal muscles, as well as beneath the scapula and serratus anterior muscle [23]. Subsequent studies have demonstrated wider (T3 to T12) dermatomal coverage in the RISS block [24]. It is generally indicated for patients undergoing abdominal surgeries, including laparoscopic procedures, open cholecystectomy, and hernia repairs, when effective postoperative acute pain control is crucial [12, 13].

In a proof-of-concept study comprising cadaveric and retrospective case series elements, 15 patients who underwent abdominal surgery, experienced rib fractures, or suffered from chest tube or postoperative incisional chest wall-related pain received a RISS block. The research indicated a reduction in cold temperature feeling from T2 to T12 [23].

The variability observed in dermatomal coverage is attributed to the levels of local anesthetic injection in the block. In the present study, the rhomboid intercostal part of the block was administered at level of the 5th vertebra, while the sub-serratus component was performed at the T8 level, resulting in confirmed analgesia in the T6-T10 area. This study extended the potential indications for the RISS block to include open radical nephrectomy via subcostal incision. Although the RISS block demonstrated slightly lower effectiveness compared to the ESPB, patients who received RISS experienced adequate pain control and morphine consumption. At various postoperative time points, pain scores were comparable between the two techniques.

This study likely represents the most comprehensive series to date comparing ESPB and RISS in patients undergoing radical nephrectomy for renal malignancies. Regional block techniques were administered by experienced anesthesiologists at a major tertiary care center. However, some limitations of our study should be acknowledged. Although both ESPB and RISS provided effective analgesia, we did not perform dermatomal mapping. This limits interpretation of ESPB’s early advantage, which may be attributable to its paravertebral spread. Pain beyond the initial 24-hour postoperative period was not assessed. Furthermore, the single-center design, small sample size, and use of single-shot blocks constrain the generalizability of our findings. Notably, while ESPB was associated with a statistically significant reduction in opioid consumption, the magnitude of this difference was modest. Overall, both techniques demonstrated comparable analgesic profiles. To better clarify their clinical implications, larger multicenter studies with extended follow-up are warranted.

## Conclusion

Ultrasound-guided ESPB appears to be marginally more efficient than RISS as an effective postoperative analgesic technique in patients undergoing open radical nephrectomy via subcostal incision for renal malignancies. ESPB showed a greater reduction in postoperative opioid consumption and longer duration of analgesia compared to RISS. Both techniques provided analgesia in the T6-T10 area.

## Supplementary Information

Supplementary Material 1.

Supplementary Material 2.

## Data Availability

Data generated and analyzed during the current study is available on reasonable requests for one year.

## Abbreviations

RISS: Rhomboid Intercostal Sub-serratus block
ESPB: Erector Spinae Plane Block
RCC: Renal Cell Carcinoma
ASA: American Society of Anesthesiology
PACU: Post Anesthesia Care Unit
MAP: Mean arterial blood pressure
HR: Heart Rate
VAS: Visual Analogue Scale
BMI: Body Mass Index
PCA: Patient Controlled Analgesia
PONV: Post Operative Nausea and Vomiting
LA: Local Anesthetics
