# Supplementary material for: Ultrasound-guided erector spinae plane block versus rhomboid intercostal sub-serratus plane block for postoperative analgesia in open radical nephrectomy: a randomized clinical study
Source: BMC Anesthesiol. 2025 Oct 22;25:514. doi: 10.1186/s12871-025-03377-4 (PMC12542437; doi:10.1186/s12871-025-03377-4)
Supplement: Supplementary file 2 — Supplementary Material 2. [file 12871_2025_3377_MOESM2_ESM.docx]

**Declarations:**

**Ethics approval and consent to participate**

Written informed consent was obtained from each participant. Data confidentiality was strictly maintained. The study adhered to the CONSORT reporting guidelines and was conducted in compliance with national institutional ethical standards and the ethical principles of the Declaration of Helsinki. The study was registered in clinical trial. gov <https://clinicaltrials.gov/study/NCT05822011> , trial ID (NCT05822011).

**Consent for publication**

Not applicable

**Availability of data and materials**

Data generated and analyzed during the current study is available on reasonable requests for one year.

**Competing of interests**

The authors declare that they have no competing interests.

**Funding**

There was no external funding for this work

**Authors’ contribution**

All authors read and approved the final version of the manuscript

**DA:** Contributed to interpretation and analyzation of data, shared in revising and writing the manuscript.

**SA**: Contributed to patient recruitment, data collection, shared in writing and editing the manuscript.

**AS:** Initiated the study idea, patient recruitment, interpretation and analyzation of data, shared in revising and writing the manuscript.

**MA:** Contributed to the study idea, shared in revising the manuscript.

**KE:** Contributed to the study idea, shared in revising the manuscript.

**FE:** Contributed to interpretation and analyzation of data, shared in revising and writing the manuscript.

**AF:** Contributed to interpretation and analyzation of data, shared in revising and writing the manuscript.

**WE:** Contributed to the study idea, shared in interpretation and analyzation of data, writing the manuscript.
